# Supplementary material for: Mechanical and Structural Adaptation of the Pulmonary Root after Ross Operation in a Murine Model
Source: J Clin Med. 2022 Jun 28;11(13):3742. doi: 10.3390/jcm11133742 (PMC9267924; doi:10.3390/jcm11133742)
Supplement: Supplementary file 1 [file jcm-11-03742-s001.zip › jcm-1754984-supplementary.pdf]

# TABLES

**Table S1**. Primary antibodies used in the immunohistochemical analysis.

| Antibody | Host | Reactivity | Company | Antibody diluition |
|---|---|---|---|---|
| ***α-SMA*** | Mouse | Human | Dakocytomation, Glostrup, Germany | 1:200 |
| ***vWF*** | Rabbit | Rat (mouse, human) | Dakocytomation, Glostrup, Germany | 1:200 |
| ***iNOS*** | Rabbit | Rat (mouse, human) | OriGene Technologies GmbH, Herford, Germany | 1:100 |
| ***CD45*** | Rabbit | Rat (mouse, pig, human, rhesus monkey) | Abcam, Cambridge, United Kingdom | 1:250 |
| ***CD68*** | Mouse | Rat (mouse, rabbit, human) | Abcam, Cambridge, United Kingdom | 1:250 |

α-SMA: alpha-smooth muscle actin; iNOS: inducible nitric oxide synthase; vWF: von Willebrand factor.

**Table S2**. Semiquantitative evaluation of calcium deposits.

| Animal ID | Calcification score |
|---|---|
| **LV01** | 0 |
| **LV02** | 2 |
| **LV03** | 1 |
| **LV04** | 0 |
| **LV06** | 1 |
| **LV08** | 2 |
| **LV09** | 4 |
| **LV10** | 0 |
| **LV11** | 0 |

Calcification is expressed as absence (0), point-like (1), multiple point deposits (2), multiple deposits (3), massive deposition (4).

**Table S3**. Evaluation of necrosis, inflammation and α-SMA positive cells distribution.

| Animal ID | Necrosis* | CD45 (%) | CD68 (%) | PAG tunica media α-SMA^ (%) | PAG intimal hyperplasia α-SMA^ (%) |
|---|---|---|---|---|---|
| **LV01** | 0 | 1 | 5 | 70 | 90 |
| **LV02** | 0 | 1 | 5 | 50 | 75 |
| **LV03** | 0 | 1 | 2 | 54 | 92 |
| **LV04** | 1 | 5 | 5 | 65 | 95 |
| **LV06** | 1 | 1 | 1 | 75 | 90 |
| **LV08** | 1 | 7 | 2 | 55 | 96 |
| **LV09** | 1 | 10 | 5 | 73 | 95 |
| **LV10** | 1 | 2 | 5 | 71 | 93 |
| **LV11** | 0 | 3 | 2 | 76 | 92 |

* Absence = 0; presence = 1. ^ In the aorta of the SOG, the α-SMA-positive cells percentage is always nearly 98%. PAG = pulmonary artery geaft.
